# Study on the integrated self-priming process of a vehicle-mounted self-priming pump

**DOI:** 10.1016/j.heliyon.2024.e37164

**Published:** 2024-08-29

**Authors:** Yu-Liang Zhang, Kai-Yuan Zhang, Yan-Juan Zhao, Jin-Fu Li, Shao-Han Zheng

**Affiliations:** a College of Mechanical Engineering, Quzhou University, Quzhou, 324000, China; b School of Mechanical Engineering, Hunan University of Technology, Zhuzhou, 412007, China; c College of Information Engineering, Quzhou College of Technology, Quzhou, 324000, China; d College of Mechanical Engineering, Zhejiang University of Technology, Hangzhou, 310023, China

**Keywords:** Self-priming pump, Circulatory piping system, Numerical simulation, Self-priming process, Gas-liquid two-phase distribution

## Abstract

In order to explore the self-priming characteristics of the self-priming pump at the mobile pump truck, this paper established a complete three-dimensional circulatory piping system including the self-priming pump, tank, valves, inlet pipe and outlet pipe. The UDF(User Defined Functions) was used to realize the acceleration-constant speed operation process of the impeller, thus reflecting the actual changing state of the rotational speed. Based on the VOF(Volume Of Fluid) multiphase flow model and the Realizable *k-ε* turbulence model, a coupled numerical calculation of unsteady incompressible viscous flow was conducted for its self-priming process. The results show that the self-priming process of the pump can be roughly divided into four stages: the rapid suction stage, the shock exhaust stage, the rapid exhaust period and the pump residual gas discharge stage. The proportion of each stage in the total self-priming time showed an increasing trend. During the rapid suction stage, the water level in the vertical section of the inlet pipe showed a slow and then fast-rising pattern. During the shock exhaust stage, the average gas-phase volume fraction in the volute is lower than that of the impeller, and the gas content at the volute outlet is lower than that of the impeller inlet. The region at the inlet and outer edge of the impeller consistently experience significant energy losses.

## 1 Introduction

As a special type of centrifugal pump, self-priming pumps contain noticeable appearance features such as a large volume gas-liquid separation chamber and the reflux hole. Compared with ordinary centrifugal pumps, self-priming pumps have the function of self-priming. Only the initial filling is required, and the pump chamber can store a certain amount of water for the next start. It can be used in various applications such as emergency drainage, municipal, chemical, pharmaceutical, and irrigation [[1], [2], [3], [4]]. The most critical performance parameters for evaluating the quality of a self-priming pump include the self-priming height and the self-priming time. Researchers have carried out numerical simulations and experimental investigations on them in various aspects. In the numerical simulation and structural improvement of the self-priming pump startup process, Wang et al. [5] simulated the unsteady gas-liquid two-phase flow during the self-priming process of a self-priming pump. Based on the computational results, they divided the entire process into three parts. The first stage was the suction stage under the work done by the impeller; the second stage was the suction stage under the action of gas-liquid mixing and gas-liquid separation; and the third stage was the suction stage when the liquid entered the self-priming pump. The second of these stages was the main stage of the self-priming process, which determined the duration required to complete the self-priming. Wang et al. [6] also trimmed the outlet diameter of the self-priming pump impeller. The results showed that the optimum efficiency point of the self-priming pump converged to a smaller flow condition after trimming. Under the design flow condition, after a 6 % modification of the impeller profile, there was a slight decrease in hydraulic performance and a slight increase in self-priming time. However, there were improvements in the pressure pulsation amplitude and radial stresses on the volute wall. Li et al. [7,8] numerically simulated the start-up process of a self-priming pump by using the VOF multiphase flow model and slip grid technique. The simulation results indicated that during the initial self-priming stage, significant transient effects were observed in the velocities of the impeller, gas-liquid separation chamber inlet, and the sections of the volute. At the same time, they analyzed that the main reason for the oscillation of the gas content curve at monitoring points inside the impeller was the continuous generation and collapse of bubbles within the impeller. Yuan et al. [9] conducted unsteady numerical simulations of the self-priming process for a two-stage self-priming pump. The simulation results indicated that the static pressure fluctuations in the impeller of the first stage were significant during the self-priming process. As the liquid content in the gas-liquid mixture increased, the impeller's efficacy gradually strengthened. Zhang et al. [10,11] improved the efficiency of the self-priming pump significantly by making modifications to the volute shape, sealing gasket, and blade shape while ensuring good self-priming performance. Xia et al. [[12], [13], [14]] investigated the relationship between different reflux hole areas, different opening positions, and the self-priming performance of a self-priming pump using numerical simulation methods. The results indicated that larger reflux holes were beneficial in reducing the self-priming time. However, excessively large reflux hole areas would increase volume losses under high flow conditions. During the normal operating phase, the size and position of the reflux hole had a minimal impact on hydraulic performance. Specific self-priming pumps typically had an optimal reflux hole size and opening position.

In the experimental investigation of the self-priming pump startup process, Zhang et al. [15] conducted experiments to study the effects of different startup speeds on the performance of the self-priming pump when conveying water. The experimental results showed that all hydraulic performance parameters were affected by different starting speeds, with the degree of influence ranging from highest to lowest: flow rate, rotational speed, and head. Qian et al. [16] conducted visual tests on the self-priming process of a self-priming pump, revealing the transient gas-liquid changes during the self-priming process. The results indicated that the self-priming ability of the pump mainly depends on the gas-liquid mixing rate, the rate of gas-liquid two-phase flow from the volute to the gas-liquid separation chamber, and the gas-liquid separation rate. Qian et al. [17] also compared the two methods of experimental testing and numerical calculations to investigate the startup process of the self-priming pump. The comparison results showed that the water level changes in the numerical simulation were in good agreement with the experimental results. In the last stage of self-priming, the bubbles in the outlet pipe mainly included bubbly flow and slug flow.

The following three problems can be found in numerical calculation studies: Firstly, existing studies commonly used the self-priming pump itself or added a section of pipeline to the inlet and outlet as the computational model. Secondly, the inlet pipe was calculated by artificially giving certain gas-liquid distribution conditions. Thirdly, the rising phase of the impeller speed was ignored, and the constant speed case was calculated directly. Such computations inevitably led to significant discrepancies from the actual situation and failed to accurately and effectively reflect the self-priming process characteristics of the pump. In order to solve the above problems, this paper established a circulatory piping system including components such as the self-priming pump, valve, tank, and pipes. A certain amount of water was stored in the tank and the self-priming pump, and the tank level was connected to the atmosphere. Additionally, the rising stage of the rotational speed was also modeled through user-defined functions. Therefore, the computational physical model and the variation characteristics of rotational speed in this paper were consistent with the actual situation, which can effectively reflect the real self-priming process of the self-priming pump. In this paper, the calculation model was established based on the self-priming pump. After verifying the reliability of the numerical calculation method, the original geometric system was simplified to some extent to reduce the amount of calculation. Based on the simplified model, numerical calculations were carried out to reveal the changing tendency of internal flow and external characteristics of the vehicle-mounted self-priming pump in the whole self-priming process. At the same time, the internal flow of the pump in the self-priming process was analyzed by introducing vortex identification, entropy production theory, and energy gradient theory.

## 2 Numerical calculation method

### 2.1 Initial computational model and grid

**Fig. 1:**
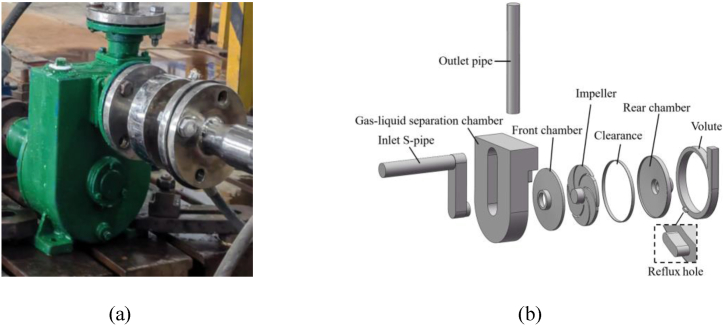
Self-priming pump (a)The physical diagram (b)Calculation domains.

The computational model in this paper is a split-type externally mixed self-priming pump with model number DKS18-40-3. The physical appearance of the pump is shown in Fig. 1(a). The three-dimensional model of the computational domain of the self-priming pump internal flow is shown in Fig. 1(b). Its main parameters are shown in Table 1.

**Table 1:** Main parameters of the self-priming pump.

Design Parameters	Symbol/Unit	Value
Rotation speed	*n*/(r/min)	2900
Flow rate	*Q*_e_/(m^3^/h)	15
Head	*H*_e_/(m)	32
Specific speed	*n*_s_	51

**Fig. 2:**
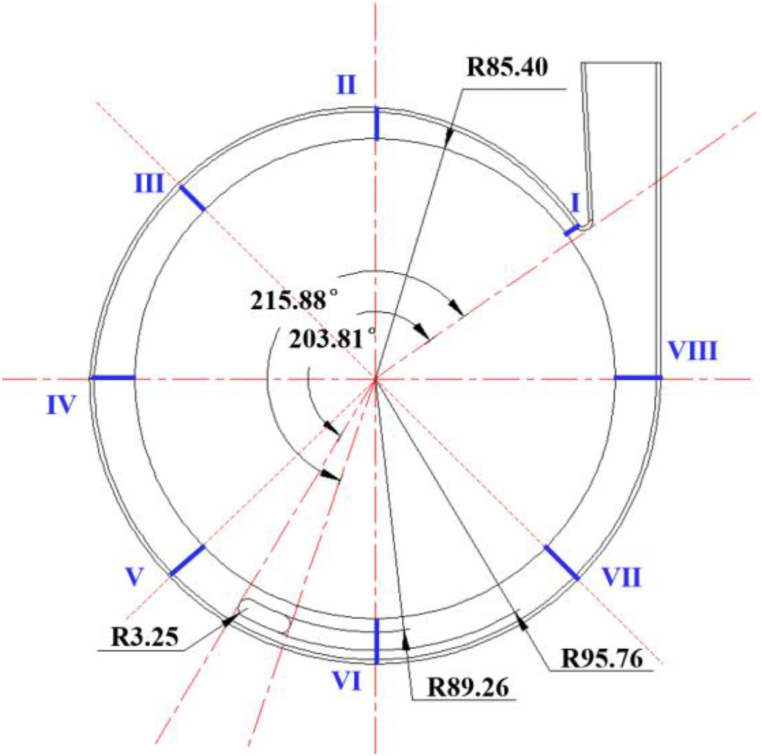
Size and location of the reflux hole.

The primary overflow components of this self-priming pump include the inlet S-pipe, the impeller, the volute, the gas-liquid separation chamber, the front chamber, the rear pump chamber and the outlet pipe. The domain of the reflux hole connects the domain of the volute to the bottom of the gas-liquid separation chamber, and the reflux hole is located at a position ranging from 203.81° to 215.88° from the tongue of the volute along the rotation direction of the impeller, as shown in Fig. 2. And eight sections are defined on the flow path of the volute, section I on the volute is located at the tongue, and the angle between sections II-VIII are all 45°.

**Fig. 3:**
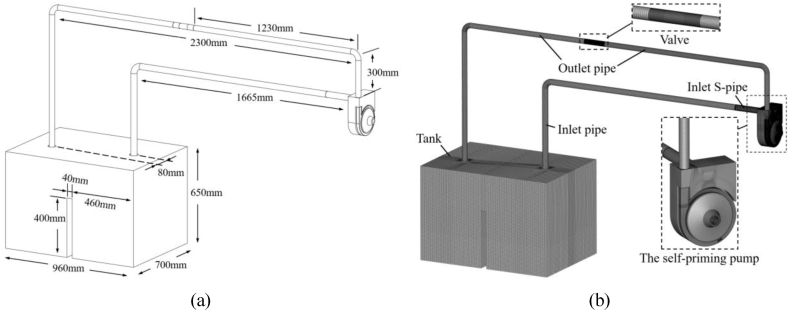
Circulatory piping system (a) Computational model (b) Computational grid.

Fig. 3(a) is the established original computational model, and the main geometrical dimensions are shown in Table 2. The piping system consists of the tank, the inlet pipe, the inlet S-pipe, the self-priming pump, the valve and the outlet pipe. The inlet pipe is connected to the inlet S-pipe, and the center distance between the inlet pipe and the outlet pipe in the direction of the x-axis is 80 mm. The water tank has a much larger storage capacity than the total volume of other components, ensuring an adequate water supply for the self-priming process. The computational domain is gridded using ANSYS ICEM as shown in Fig. 3(b). Due to the small size of the geometrical features of the inlet S-pipe, unstructured tetrahedral grid are used to ensure a high grid quality. The rest of the overflow components are divided by structured hexahedral grid. During the gridding process, the near-wall area and the area where the flow field changes more dramatically are subjected to local grid refinement. In addition, to reduce the size error, the front and back chambers of the pump, as well as the clearance domains between the impeller and volute, are merged into a single unit for meshing. The valve in the system is modeled simplified according to the working principle of the ball valve. The valve computational domain is divided into three sections: inlet section, center section and outlet section, and different valve openings are achieved by rotating the center section at different angles. In this paper the valve is always at full opening degree.

**Table 2:** Geometric dimensions of the circulatory piping system.

System Components	Value
The inlet pipe diameter	40 mm
Horizontal length of the inlet pipe	1665 mm
Vertical length of the inlet pipe	1050 mm
The outlet pipe diameter	35 mm
Horizontal length of the outlet pipe	2300 mm
Vertical length of the outlet pipe	1388 mm
Tank	960 mm × 700 mm × 650 mm

**Fig. 4:**
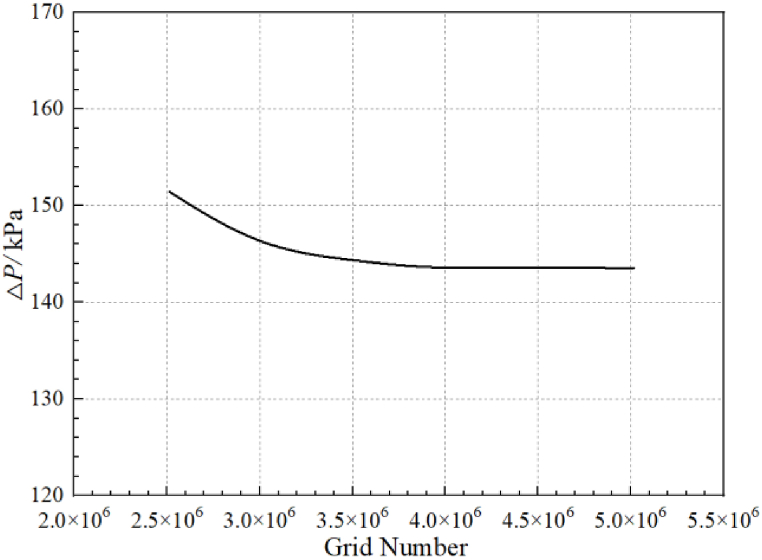
Verification of system grid independence.

The computational model of this paper is the entire circulatory piping system, so it is necessary to verify the grid independence of the entire system. The static pressure difference values at the pump outlet and the inlet of the inlet S-pipe are selected for monitoring. Fig. 4 shows that the difference in static pressure at the two overflow sections has basically stabilized when the total number of system grids is about 3,914,245. It indicates that the influence of the grid number on the results of numerical calculations is already minimal once the grid number reaches that level, so the total grid number of the system determined in Fig. 3(b) is 3,914,245. The number of grids for the tank, the inlet pipe, the outlet pipe and the valve are 870,892, 264,788, 451,416 and 465,328, respectively. The total number of grids for each overflow element of the self-priming pump is 1,861,821.

### 2.2 Governing equation

#### 2.2.1 VOF two-phase flow model

In the existing studies, Zhou et al. have confirmed that the VOF (Volume of Fluid) [[18], [19], [20]] two-phase flow model demonstrates good applicability in tracking and capturing the free surface within centrifugal pumps and pipelines, as well as accurately calculating the volume fraction of the water phase. The numerical calculation [18,19] results are in good agreement with the experimental results. Therefore, this paper simulates the gas-liquid two-phase flow during the startup of the self-priming pump based on the VOF model. The whole self-priming process of the pump is a gas-liquid two-phase mixed complex flow, not a uniform flow. And the whole simulation process is isothermal flow, do not take into account the influence of thermal effects. The basic equations of the VOF model for transient calculations include the volume fraction continuity equation, the continuity equation, and the momentum equation, which are of the following forms:

The volume fraction continuity equation:

(1) $$\frac{\partial \alpha_{1}}{\partial t}+u\cdot \nabla \alpha_{1}=0$$

(2) $$\frac{\partial \alpha_{2}}{\partial t}+u\cdot \nabla \alpha_{2}=0$$

The continuity equation:

(3) $$\frac{\partial \rho }{\partial t}+\nabla \cdot \left(\rho u\right)=0$$

The momentum equation:

(4) $$\frac{\partial \rho u}{\partial t}+\left(\rho u\cdot \nabla \right)u=-\nabla p+\nabla \cdot \left[\mu \left(\nabla u+\nabla u^{T}\right)\right]+\rho g+F$$

where: $\alpha_{1}$ and $\alpha_{2}$ represent the volume fractions of the water and gas phases, respectively, and $\alpha_{1}+\alpha_{2}=1$; u is the velocity; *t* is the time; *p* is the static pressure; ∇ is the differential operator; μ is the kinetic viscosity coefficient of the mixture, $\mu =\alpha_{1}\mu_{1}+\alpha_{2}\mu_{2}$, $\mu_{1}$ and $\mu_{2}$ are the kinetic viscosity coefficients of the water and gas phases, respectively; ρ is the density of the mixture, $\rho =\alpha_{1}\rho_{1}+\alpha_{2}\rho_{2}$, $\rho_{1}$ and $\rho_{2}$ are the densities of the water and gas phases, respectively; ***g*** is the acceleration of gravity; ***F*** is the equivalent volumetric force form of surface tension [21].

#### 2.2.2 Turbulence model

The Realizable *k-ε* two-phase equations model adopts a new turbulent viscosity formulation, so it meets the constraints of the Reynolds stress and remains consistent with the real turbulence in terms of the Reynolds stress. Zhang et al. [10,21] numerically investigated the gas-liquid two-phase flow inside the impeller of a submersible pump based on the Realizable *k-ε* turbulence model in combination with the Eulerian-Eulerian multiphase flow model and obtained highly reliable results. It is proved that the Realizable *k-ε* turbulence model is suitable for the simulation of the self-priming pump startup process, so the Realizable *k-ε* turbulence model is used in this paper to close the set of control equations. The constraint equations for the turbulent kinetic energy *k* and the turbulent dissipation rate *ε* in this turbulence model are as follows:

(5) $$\frac{\partial \left(\rho k\right)}{\partial t}+\frac{\partial \left(\rho ku_{j}\right)}{\partial x_{j}}=\frac{\partial }{\partial x_{j}}\left[\left(\mu +\frac{\mu_{t}}{\sigma_{k}}\right)\frac{\partial k}{\partial x_{j}}\right]+\rho \left(P_{k}-\varepsilon \right)$$

(6) $$\frac{\partial \left(\rho \varepsilon \right)}{\partial t}+\frac{\partial \left(\rho \varepsilon u_{j}\right)}{\partial x_{j}}=\frac{\partial }{\partial x_{j}}\left[\left(\mu +\frac{\mu_{t}}{\sigma_{\varepsilon }}\right)\frac{\partial \varepsilon }{\partial x_{j}}\right]+\rho C_{1}E\varepsilon -\rho C_{2}\frac{\varepsilon^{2}}{k+\sqrt{v\varepsilon }}$$

where the coefficient $C_{1}=\max\left(0.43,\frac{\eta }{\eta +5}\right)$, coefficient of eddy viscosity $C_{\mu }=\frac{1}{A_{0}+A_{s}U^{*}k/\varepsilon }$. In the equation for the turbulent turbulent eddy viscosity coefficient, $A_{s}=\sqrt{6}\cos\;\phi$, $\phi =\frac{1}{3}\cos^{-1}\left(\sqrt{6}W\right)$, $W=\frac{E_{ij}E_{jk}E_{ki}}{{\left(E_{ij}E_{ij}\right)}^{\frac{1}{2}}}$, $U^{*}=\sqrt{E_{ij}E_{ij}+{\tilde{\Omega }}_{ij}{\tilde{\Omega }}_{ij}}$, ${\tilde{\Omega }}_{ij}=\Omega_{ij}-2\varepsilon_{ijk}\omega_{k}$, $\Omega_{ij}={\bar{\Omega }}_{ij}-\varepsilon_{ijk}\omega_{k}$. The constant values in the above equation are: *σ*_*ε*_ = 1.2, *C*_2_ = 1.92, *A*_0_ = 4.0. The time derivative is zero when calculated as a constant.

### 2.3 Numerical calculation methods

In the self-priming process of the pump, there is an unsteady gas-liquid two-phase flow inside, assuming the following [22]: Gas and liquid phases have constant physical properties, with the liquid phase being incompressible and the gas phase considered as a continuous medium; The self-priming process is considered to be completed when the average volume fraction of the gas phase in the entire pump chamber is less than 2 %; No heat exchange occurs in the medium during the self-priming process.

In this paper, numerical calculations of the self-priming transient process are performed based on the commercial software FLUENT 21.1. During the iterative calculations, the convergence accuracy of the velocity components in each direction, the turbulent kinetic energy *k*, and the turbulent dissipation rate *ε* are set to default values of 1 × 10^−4^. Data transfer between different domains is achieved by interface coupling to ensure continuity throughout the entire system. The coupling of pressure and velocity fields is solved by the SIMPLEC algorithm. No-slip boundary conditions are used at the solid wall, and the standard wall function is used at the near-wall to solve the flow calculation problems in the near-wall region and the low Reynolds number case.

In the self-priming process, the rotational speed of the impeller of the self-priming pump rises rapidly from a static state to a stable value and maintains constant speed operation. In order to more realistically simulate the self-priming process, the impeller speed change process is divided into two stages: accelerating rising stage and constant speed operation. The control of impeller speed is realized by loading user-defined functions, as shown in Equation (7).

(7) $$n=\left\{ \begin{array}{c} 14500t\;t\;<\;0.2\\ 2900\;t\geq 0.2 \end{array}\right.$$

where *n* is the transient rotational speed of the impeller and *t* is the time.

In order to save computational cost, the numerical simulations in this paper are calculated with variable step sizes. Before 0.20 s, the time step is set to 0.00025 s; After 0.20 s, the impeller operates at a stable rotational speed, and the time step is set to 0.0025 s.

**Fig. 5:**
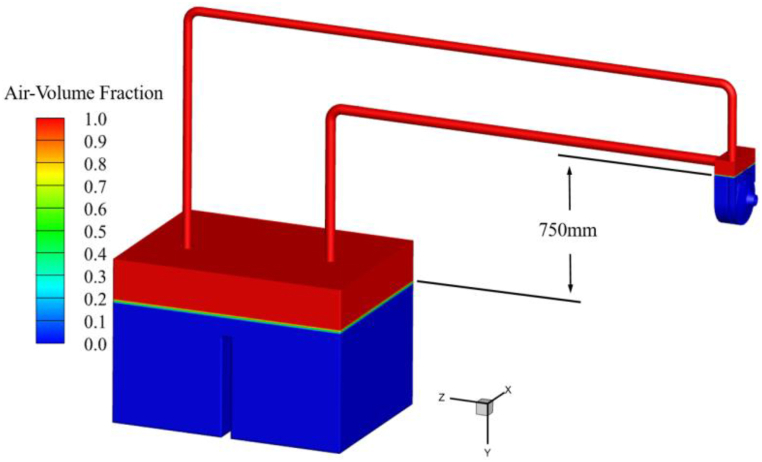
Initial state of gas-liquid two-phase.

The initial state of the gas-liquid two-phase of the circulatory piping computational model is shown in Fig. 5. In order to ensure that the boundary conditions of the computational model are consistent with the experimental setup, atmospheric pressure is set at the top of the water tank. Other than that, there are no other boundary conditions, and the data transfer between each overflow component is realized by coupling calculation. The vertical distance between the liquid level in the tank and the initial water level in the pump is 0.75 m. The average gas volume fraction in the upper part of the tank is 1, and the average liquid volume fraction in the lower part is 1.

### 2.4 Reliability verification of numerical methods

**Fig. 6:**
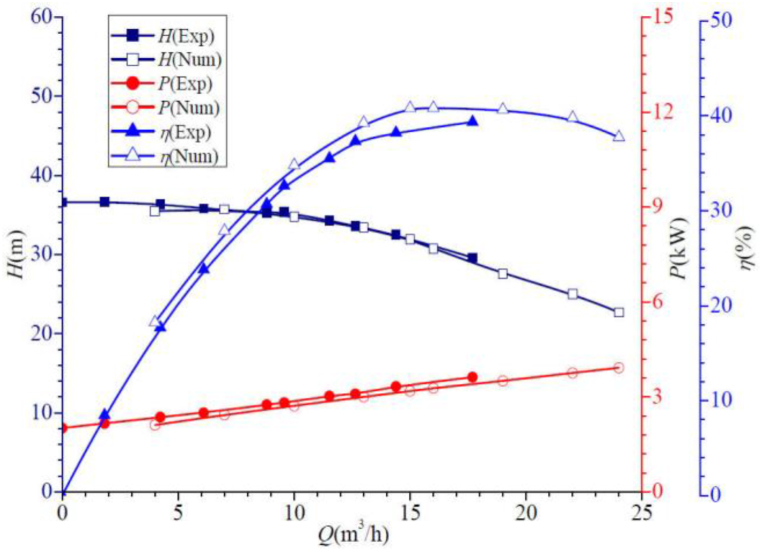
Comparison of external characteristics of the self-priming pump.

The numerical prediction results of the external characteristics of the self-priming pump are compared with the experimental results as shown in Fig. 6, which shows that overall the two are in good agreement. As the flow rate increases, the experimental head and calculated head are gradually reduced. When operating near the rated flow rate, the head error between the two is very small. In the large and small flow conditions, the error is slightly larger, and the difference is about 0.862 m, the error is about 2.372 %. The shaft power increases gradually with the flow rate increase, similar to the error observed in the head curve. Under the small and large flow conditions, the error between the test values and the calculated values is large, and the maximum difference of the shaft power is about 0.229 kW, with an error of about 9.778 %. The calculation results of hydraulic efficiency also basically match with the test results, the curves show the process of rising and then falling, there is a maximum efficiency value, which occurs near the design flow conditions, and the error between the calculation results and the test results is within 8 %. In conclusion, the simulation errors are within the acceptable range by using the above numerical calculation method, so the above numerical calculation method has high reliability.

### 2.5 Model simplification

**Fig. 7:**
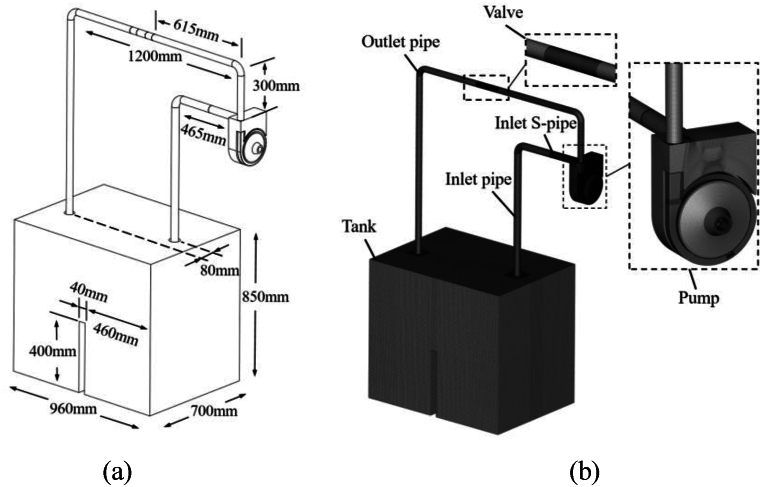
Simplified circulatory piping system (a) Computational model (b) Computational grid.

**Fig. 8:**
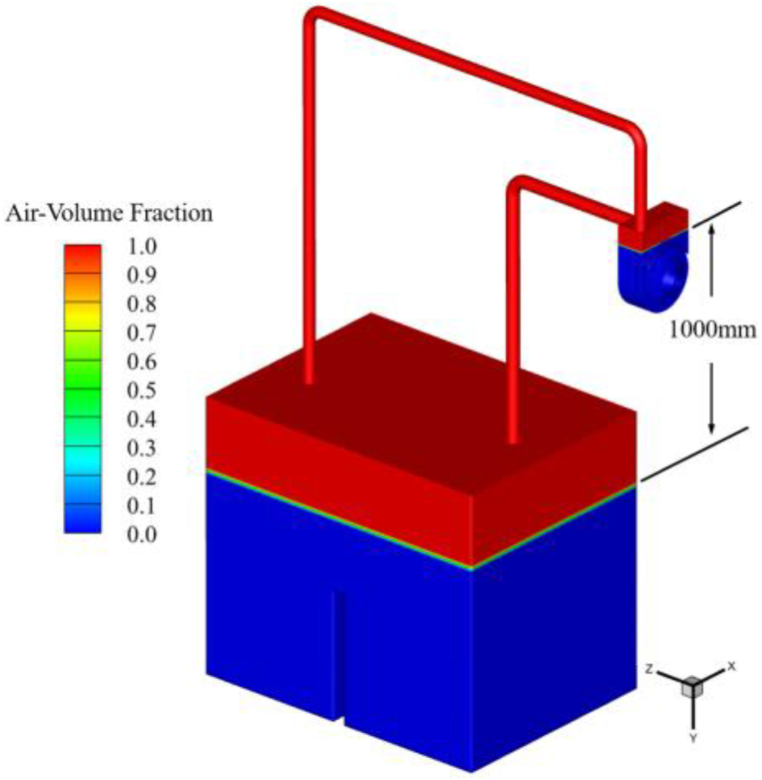
The initial state of the simplified model gas-liquid two-phase.

In the above calculation process, the authors of this paper found that when the calculation model shown in Fig. 3 is used, there is a large computational workload for the self-priming process, which results in a very long computation time. The reason for this is that the length of the water inlet pipe is too long, and the volume of air stored before the self-priming is too large (as shown in Fig. 5), resulting in a vast computational workload to complete the entire self-priming process, and the time required is very long. Therefore, it is necessary to simplify the calculation model shown in Fig. 3. The inlet and outlet pipes are shortened to appropriate lengths, while the height of the tank is increased, and the self-priming height is set to 1.0 m. The simplified computational model dimensional information is shown in Fig. 7(a). Fig. 7(b) shows the simplified computational system grid. After simplification, the total number of grids is less different than before, totaling 3,903,026 grids. The numbers of grids for the tank, the inlet pipe, the outlet pipe, and the valve are 1,021,209, 223,856, 330,812, and 465,328, respectively. The total number of grids for each overflow component of the self-priming pump is 1,861,821, which remains exactly the same as before simplification. After simplification, the previously validated numerical calculation method is used to complete the unsteady flow numerical calculation of the complete self-priming process of the pump. The initial state of the gas-liquid two-phase in the simplified calculation model is shown in Fig. 8.

## 3 Result analysis

### 3.1 Changes of external performance

**Fig. 9:**
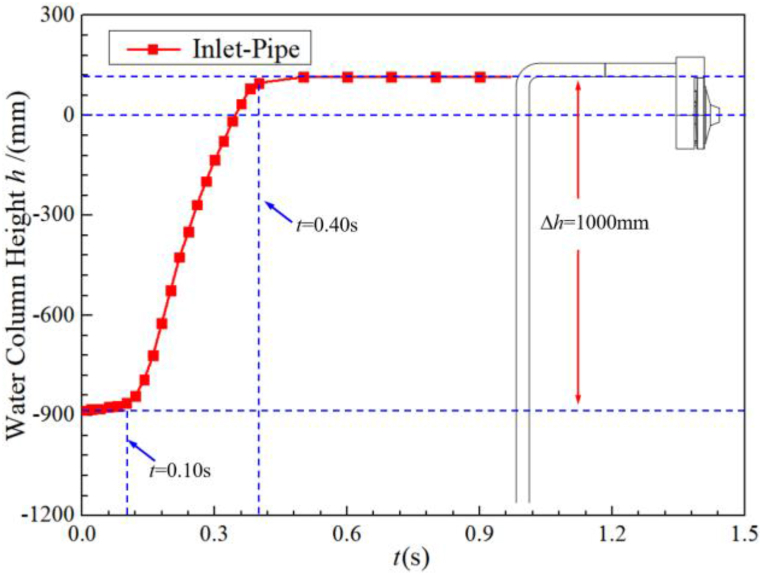
Water level changes in the inlet pipe during self-priming process.

During the self-priming process, the internal flow of the self-priming pump is very complex. The fluid inside the pump undergoes a series of transformations. It starts from an initial state where the gas-liquid interface is clear, gradually turns into a complex flow state of gas-liquid mixing, and then gradually separates inside the gas-liquid separation chamber. Finally, the self-priming process is completed and the pump is entirely occupied by the liquid phase. Fig. 9 shows the evolution of the rising height of the liquid column with time in the vertical section of the inlet pipe in the yoz plane (x = 0). It can be seen that there is a more pronounced accelerated rising stage during the water level rise. In the first stage (0.0 s < *t* < 0.10 s), the water level in the inlet pipe rises very slowly, only 21.6 mm. This is mainly because the water body in the whole system in the first stage is static and has a large inertia. The rotational speed of the impeller in this stage is relatively low, and the vacuum degree caused by the rotation of the impeller is also relatively low. In the second stage (0.10 s < *t*< 0.40 s), the water level in the inlet pipe rises rapidly. At *t*= 0.40 s, the water level rises by 982.1 mm near the inlet S-pipe. This is because the rotational speed of the impeller first accelerates to 2900 r/min to complete the acceleration process and then remains constant at this speed during this stage. Therefore, the vacuum level in the pump is higher, resulting in an accelerated rise in water level. After 0.40 s, it enters the third stage, when the water level gradually rises to the horizontal part of the inlet pipe and then enters the pump through the inlet S-pipe.

**Fig. 10:**
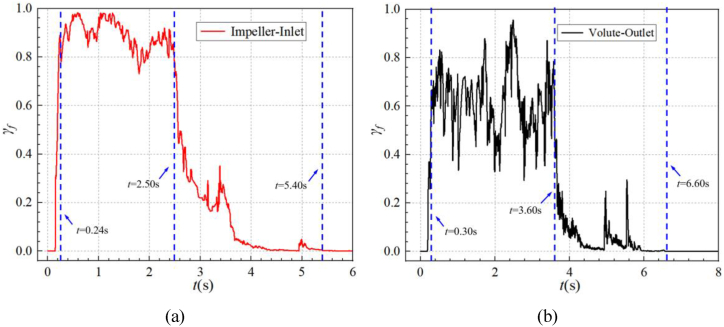
Gas content in cross-section during self-priming process (a)impeller inlet (b)volute outlet.

**Fig. 11:**
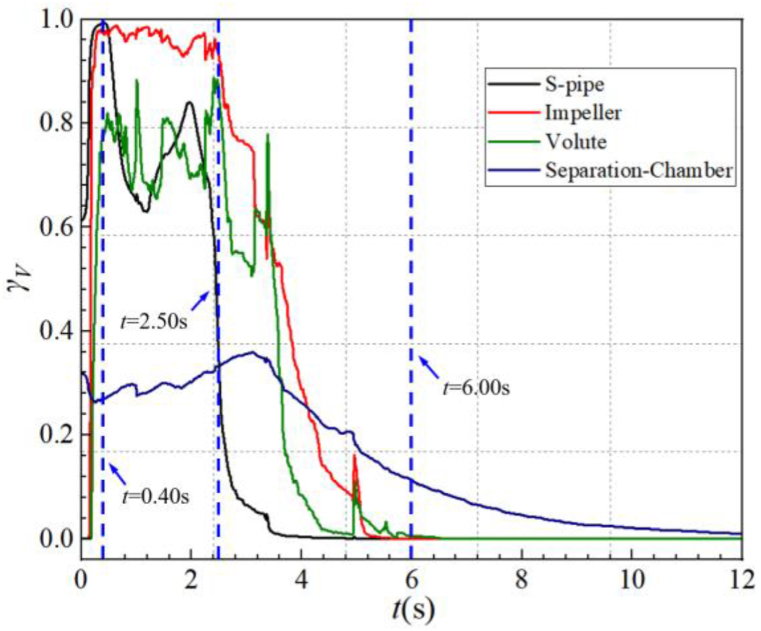
The gas-phase volume average fraction of each over-flow component.

To reveal the evolution process of the gas-liquid two-phase flow within the main overflow components of the self-priming pump, Fig. 10 shows the curve of gas content (γ_*f*_) with time at the impeller inlet and volute outlet during the self-priming process. Fig. 11 shows the curve of the average gas volume fraction (*γ*_*V*_) in the cavity of each over-flow component with time during the self-priming process.

**Fig. 12:**
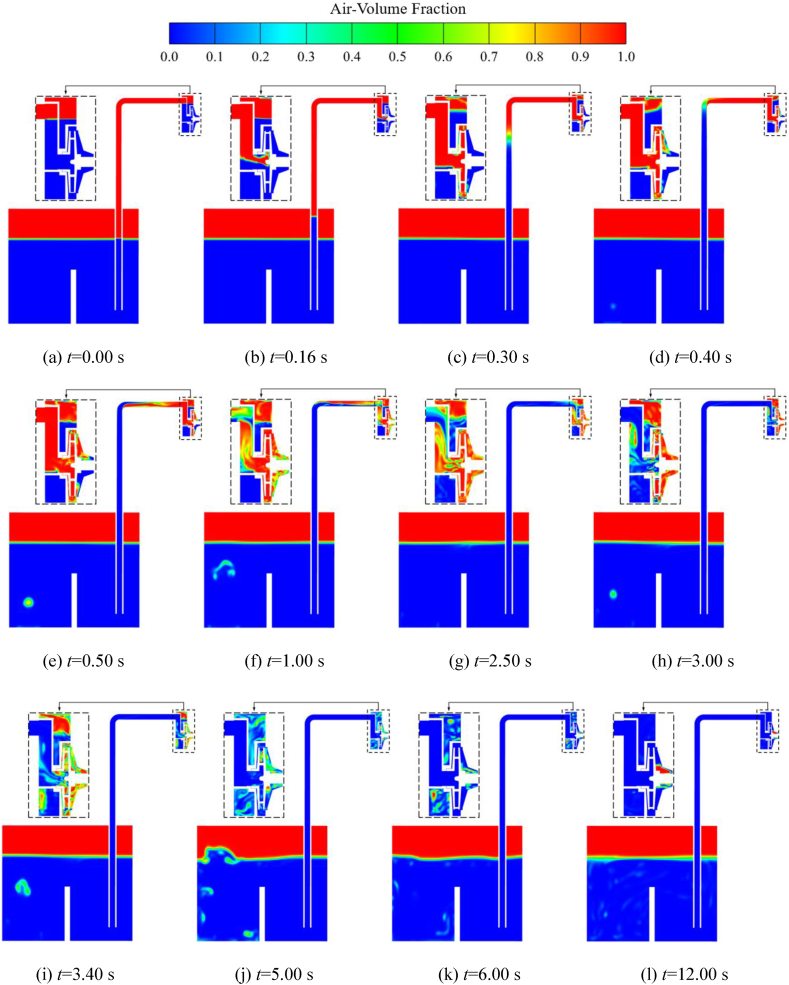
Gas-liquid two-phase distribution in the inlet pipe cross-section during the self-priming process.

Combining Fig. 10(a) and (b), the gas content curves on the impeller inlet and volute outlet cross-sections can be roughly divided into four stages. However there are slight differences in the time nodes. Fig. 10(a) shows that in the first stage (*t*< 0.24 s), the impeller is accelerated to a rated speed of 2900 r/min, and part of the liquid in the volute chamber is discharged into the gas-liquid separation chamber and the outlet pipe, while the liquid in the inlet S-pipe enters the impeller. Subsequently, under the action of atmospheric pressure, the gas in the inlet pipe is also sucked into the impeller, so the gas phase content at the impeller inlet rises sharply from zero value to a higher level. During the second stage (0.24 s < *t*< 2.50 s), the gas content curve fluctuates at a higher level, always accompanied by a larger value fluctuation. At about t = 0.60s, the gas phase content at the impeller inlet reaches the maximum value, of about 97.91 %. In the third stage (2.50 s < *t* < 6.00 s), the gas phase content at the impeller inlet decreases rapidly, which is attributed to the fact that the liquid in the inlet pipe has reached the impeller inlet at *t* = 2.50 s, and the gas is gradually discharged. At about *t* = 3.40 s and *t* = 5.00 s, there is a significant increase in the gas phase content, corresponding to 33.81 % and 4.35 % of the transient gas phase content, respectively. Combining Fig. 10, Fig. 12, it can be found that the sudden increase in the gas content curve at *t* = 3.40 s is because some of the gas remaining in the inlet S-pipe and in the front pump chamber is sucked into the impeller chamber at this time; At *t* = 5.00 s, the second increase is caused by the fact that part of the gas in the gap in the front pump chamber is sucked into the impeller chamber because the average gas volume fraction in the inlet S-pipe is very close to the zero value at this moment. Then the residual gas is discharged through the outlet of the pump after mixed with the two-phase flow in the volute. In the fourth stage (*t* > 6.00 s), it can be found from Fig. 10(a) that the gas-phase content of the impeller inlet has basically dropped to nearly zero, indicating that the impeller inlet has been entirely occupied by the liquid in the inlet pipe.

Fig. 10(b) shows that the gas content variation curves at the volute outlet during the self-priming process can also be roughly divided into. Compared with Fig. 10(a), it can be seen that in terms of the time when the characteristic change occurs, the volute outlet lags more obviously behind the impeller inlet. This is because after the system is turned on, the gas sucked into the impeller mixes with liquid, subsequently being discharged into the volute along the impeller channel under the action of centrifugal force, with a specific time interval. In the first stage (*t* ≤ 0.30 s), the surface gas content at the volute outlet rises to a high level. This is because the gas inside the impeller has started to mix with the liquid and be discharged from the volute. In the second stage (0.30 s < *t* ≤ 3.60 s), the gas content at the outlet of the volute exhibits significant fluctuations at a high level. However, the average value is lower than that at the inlet of the impeller. This same pattern is also observed in the average gas volume fraction shown in Fig. 11. At this stage, the average gas-phase volume fraction in the volute is lower than that of the impeller, and the gas content at the volute outlet is lower than that of the impeller inlet. This is because the liquid in the inner wall of the volute can not be completely discharged in time. And during this stage, most of the impeller and the volute are in gas phase. The gas inside the volute continues to move along the flow path. As a result, the pressure at the bottom of the volute flow path is small, while there is always a large amount of liquid at the bottom of the gas-liquid separation chamber. Under the action of pressure difference between the two and the gravity of liquid, the liquid at the bottom of the gas-liquid separation chamber gradually enters the volute through the reflux hole to participate in gas-liquid mixing. During the third stage (3.60 s < *t* ≤ 6.60 s), the gas content at the volute outlet decreases rapidly. During this period, there are two noticeable increases. Similar to the impeller inlet, these increases are also due to the residual gas in the inlet S-pipe and the front pump chamber being gradually discharged. In the fourth stage (*t* > 6.60 s), the gas content basically drops to zero value. This indicates that the gas phase inside the volute and the impeller has been completely discharged into the gas-liquid separation chamber and the outlet pipe after that.

**Fig. 13:**
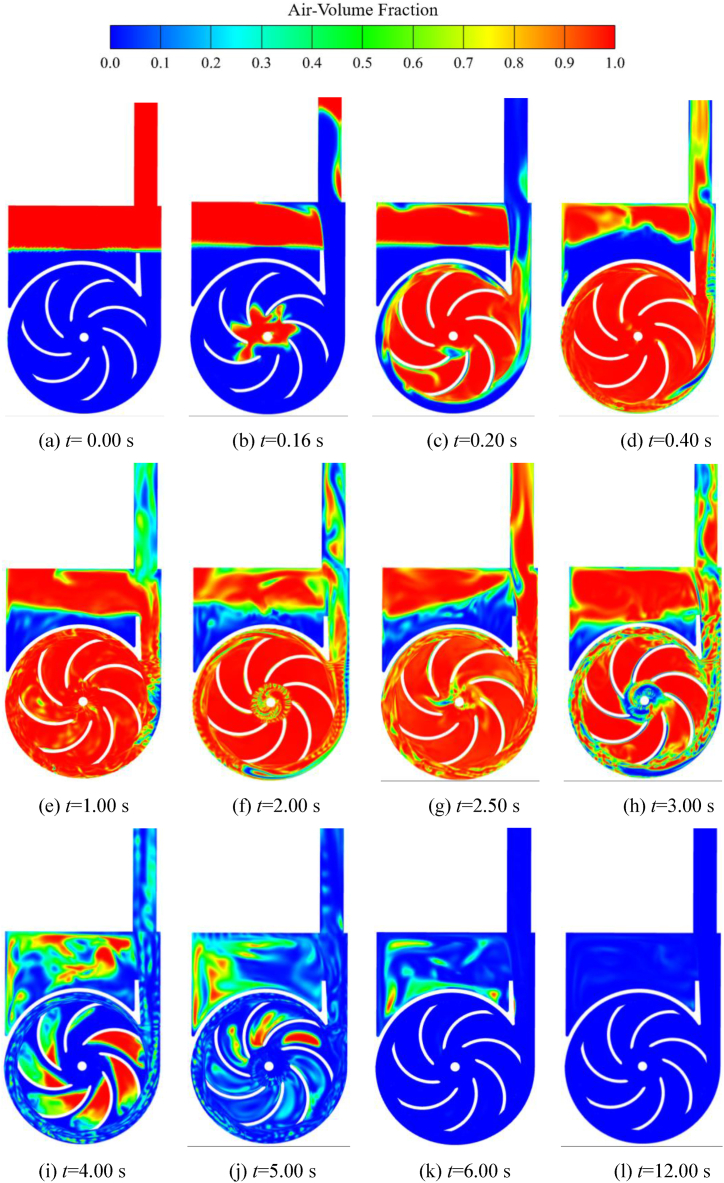
Gas-liquid two-phase distribution of cross section in impeller during the self-priming process.

Combining Fig. 10, Fig. 11, it can be observed that, apart from the gas-liquid separation chamber, the changes in the average gas volume fraction curve in the other flow components can be divided into four stages. The first stage (*t* < 0.40 s) is the rapid suction stage. The average gas volume fraction in the inlet S-pipe, the impeller, and the volute cavities rises very rapidly. And it can be found that the volute corresponds to the end point of the curve rising process with a certain lag compared to the impeller. This is mainly because the low-pressure region of the impeller inlet prompts the liquid in the tank to move upward along the inlet pipe. Then the gas enters the inlet S-pipe, the impeller, the volute, and the gas-liquid separation chamber in turn. In *t* < 0.25 s, part of the original water in the impeller and the volute enters into the gas-liquid separation chamber, resulting in a gradually decreasing gas phase volume average fraction in the gas-liquid separation chamber. Until *t* = 0.40 s, most of the liquid in the impeller, volute and inlet S-pipe has been discharged from the volute outlet into the gas-liquid separation chamber and the outlet pipe, and the changing process of the gas-liquid distribution can be observed in Fig. 12(a)–(d) and Fig. 13(a)–(d). In the second stage (0.40 s < *t* < 2.50 s), the curves of the average gas volume fraction of the chambers are maintained at a high level overall, and there are large fluctuations. This stage is the shock exhaust stage of the self-priming pump, and it is also the most critical stage in the self-priming process of the self-priming pump. The gas at the impeller inlet is continuously sucked in and mixed with the liquid at the outer edge of the impeller. A portion of the gas-liquid two-phase flow is discharged into the gas-liquid separation chamber for phase separation, so the average gas phase volume fraction inside the gas-liquid separation chamber gradually increases overall. Another portion is directly discharged out of the pump through the outlet pipe, as shown in Fig. 13(b) and (c). In the third stage (2.50 s < *t* < 6.00 s), the average gas volume fraction decreases rapidly. At *t* = 2.50 s, the liquid has gradually entered the impeller chamber. The entry of the liquid gradually restores the work capacity of the impeller. The liquid is rapidly dumped into the volute, and the vacuum near the impeller inlet is further reduced, which causes the liquid inside the inlet pipe to be rapidly sucked into the impeller, so the average gas volume fraction inside the inlet S-pipe as well as the impeller decreases rapidly. The liquid in the impeller enters the volute along the impeller channel and continues to mix with the gas-liquid mixture, and then is discharged into the gas-liquid separation chamber and the outlet pipe along the inner wall of the volute, which is called the rapid exhaust stage in the self-priming process. At *t* = 3.10 s, the average gas volume fraction in the gas-liquid separation chamber reaches the maximum value, then enters a longer exhaust stage, and the exhaust rate gradually slows down with time. At *t* = 6.00 s, except for some residual gas in the gas-liquid separation chamber, most of the gas has been discharged from the pump. The fourth stage (*t* > 6.00 s) is discharging the residual gas in the pump. The residual gas in the gas-liquid separation chamber gradually mixes into the liquid at the volute outlet and is eventually discharged. The exhaust rate of this stage is very slow and takes the longest time.

### 3.2 Distribution of gas-liquid two phases

In order to observe more clearly the real-time changes of the gas-liquid two-phase flow throughout the circulatory system during the self-priming process, the distribution of the gas-liquid two-phase over time in the middle cross-section of the inlet pipe (yoz plane) is given in Fig. 12.

From Fig. 12(a)–(d), the evolution of the liquid level height in the inlet pipe over time can be clearly observed, this is the rapid suction stage in the self-priming process of the pump. Combining the gas content curves of the impeller inlet in Fig. 12(b) and (a), it can be found that the gas in the inlet S-pipe has already entered the impeller cavity at *t* = 0.16 s. The position of the gas into the impeller is more towards the upper part of the inlet, which is because some liquid still remains at the bottom of the inlet S-pipe; At *t* = 0.40 s, the water level has basically risen to the horizontal part of the inlet pipe; After *t* = 0.40s, the liquid in the inlet pipe moves horizontally to the pump at a slower speed, while the gas mixed with the liquid in the pump is gradually discharged; When *t* = 2.50 s, some liquid reaches near the impeller inlet and is sucked in, mixing with the gas-liquid mixture inside the pump, and then discharged from the pump outlet; From Fig. 12(k) can be seen, at *t* = 6.00 s, the gas in the impeller and the volute has basically been discharged. Afterwards, it is the stage where the remaining gas inside the pump is slowly discharged. In addition, it can be found that at *t* = 6.00 s, the gas in the front pump chamber has been completely discharged, while there is still a little gas in the rear pump chamber. This is mainly due to the interconnection between the front pump chamber, throat clearance, and the inlet S-pipe. After the gas enters the front pump chamber, it can be discharged through the gap between the throat clearance and then sucked back into the impeller chamber. While the rear pump chamber only has an inlet and the gas that enters it cannot be discharged.

Fig. 13 shows the distribution of the gas-liquid two-phase in the middle cross-section (xoy plane) of the impeller during the self-priming process of the pump. From Fig. 13(a)–(d), it can be seen that in the rapid suction stage, the gas in the inlet pipe is sucked into the impeller and is discharged along the flow path to mix with the liquid near the outer edge of the impeller, and then discharged from the volute outlet. It can also be seen that part of the gas-liquid mixture enters the gas-liquid separation chamber for the separation. Due to the significant difference in density between the gas and liquid phases, the liquid gradually sinks while the gas remains above the gas-liquid separation chamber, this is in line with the simulation results in the literatures [8,17]. The other part of the gas-liquid mixture is directly discharged through the pump outlet. Fig. 13(e)–(g) are in the shock exhaust stage of the self-priming pump. In this stage, most of the liquid within the two-phase flow in this process is provided by the residue on the inner wall of the volute and the reflux hole at the bottom of the gas-liquid separation chamber. Moreover, most of the two-phase flow in the impeller and volute is gas. Such a process is observed in the literature [17] as well. However, in the literature [3], the numerical calculations did not show a significant the shock exhaust stage. Fig. 13(g)–(k) show the rapid degassing stage, at *t* = 3.00 s, the liquid phase enters the impeller inlet. It is noteworthy that the liquid entering the impeller channel is mainly distributed on the working surface of the blades, and then it is quickly discharged into the volute. While the overall movement of the gas is relatively slow, distributed in the back of the blades and the center of the channel, mainly caused by the significantly different density of the two gas-liquid phases. Fig. 13(k) and (l) show the residual gas discharge stage of the pump, that is, the gas in the gas-liquid separation chamber is carried out by the liquid at the volute outlet, and the exhaust rate is very slow.

**Fig. 14:**
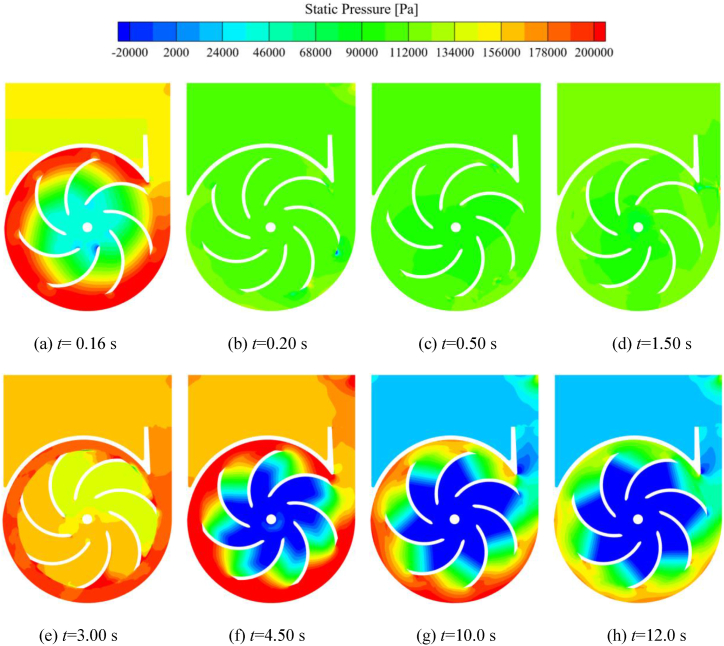
Static pressure distribution on the impeller cross-section during self-priming process.

Fig. 14 shows the static pressure distribution in the middle cross-section (xoy plane) of the impeller during the self-priming process. It can be seen from Fig. 14(a), at *t* = 0.16 s, the impeller is still in the process of acceleration, at this time, the impeller speed has reached a high level, and most of the area in the impeller is still in the liquid phase, so the impeller's overall work capacity and pressurisation capacity is strong. Therefore, at the impeller inlet, there is a low-pressure area, prompting the gas inside the inlet pipe to enter the impeller, which is manifested as a rapid suction phenomenon. As can be seen in Fig. 14(b)–(d), the impeller and volute in the majority of the gas phase, and the impeller's work capacity and the pressurisation ability is weak, so the pressure in the pump is relatively low. The pressure difference between the impeller inlet and the inside of the channel is very small, so the suction rate of the impeller in this stage is also very slow. At *t* = 3.00 s, the liquid has gradually entered the impeller, the work capacity of the impeller is gradually restored. In conjunction with Fig. 14(h) it can also be seen that the gas-liquid mixture inside the volute also has high pressure under the action of the impeller. Thereafter, as the liquid gradually occupies the impeller and volute, the impeller is more capable of doing work, and the pressurisation effect is more obvious, the vacuum at the impeller inlet is higher, so the pressure difference between the impeller inlet and the inside of the flow is larger, and the self-priming pump gradually enters into the suction-drainage operation state.

**Fig. 15:**
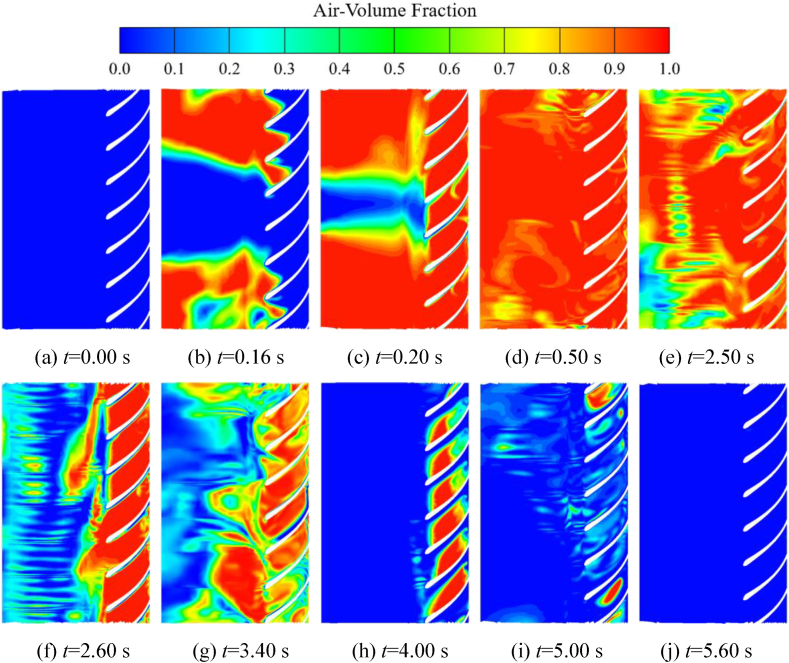
Distribution of gas-liquid two-phase in the circumferential direction of the blades during the self-priming process.

Expanding the blades of the self-priming pump in the circumferential direction can more intuitively observe the movement process of the gas-liquid two-phase inside the impeller. From Fig. 15(a)–(c), it can be seen that the gas inside the inlet S-pipe enters the impeller channel at *t* = 0.16 s, and at this time, a part of the liquid still remains at the bottom of the inlet S-pipe, which is shown in Fig. 15(b)–(c), i e., as the blue area in the middle part. Fig. 15(d) indicates that at *t* = 0.50 s, the liquid remaining in the inlet S-pipe has been sucked into the impeller, and the impeller is basically occupied by the gas phase at this time. Fig. 15(e)–(j), the liquid in the inlet pipe gradually enters the impeller under the action of atmospheric pressure at *t* = 2.50 s, resulting in a large pressure drop at the impeller inlet. The liquid in the inlet pipe is sucked in quickly, and the gas remaining in the impeller channel is wrapped up to the outer edge of the impeller for gas-liquid mixing, showing the phenomenon of rapid exhaust. After *t* = 5.60 s, most of the gas in the impeller has been discharged.

**Fig. 16:**
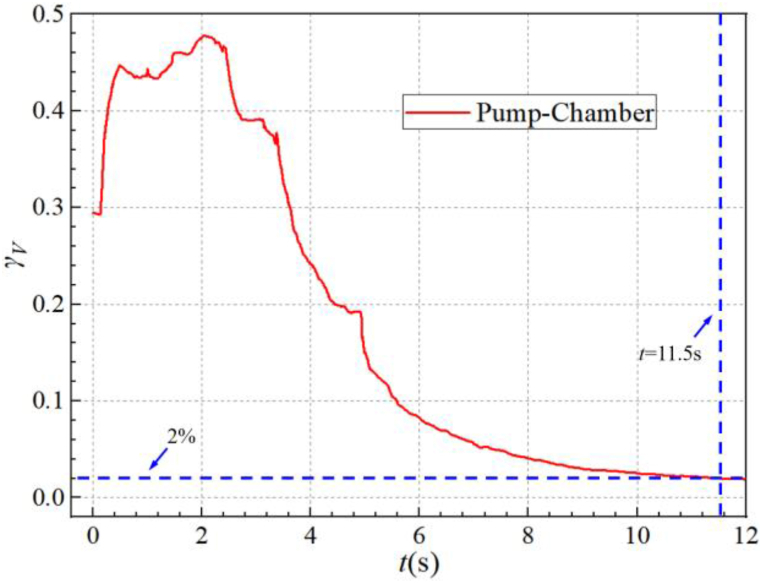
The gas-phase volume average fraction in the pump chamber during the self-priming process.

Fig. 16 shows the evolution of the gas-phase volume average fraction (*γ*_*V*_) inside the entire self-priming pump chamber over time. In theory, the completion time of self-priming for a pump is the time required for all the gas in the inlet pipe, the inlet S-pipe, and the pump chamber to be discharged entirely outside the pump. However, it is very difficult to achieve a state of complete gas discharge during the process of experimentation or numerical calculation. This is because the exhaust rate is extremely small in the final stage of self-priming. Therefore, it is considered that the self-priming of the pump is completed when the gas-phase volume average fraction in the entire pump chamber is below 2 %.

Before startup, the initial liquid level inside the pump is aligned with the bottom of the inlet S-pipe. The *γ*_*V*_ in the pump is about 29.43 % at *t* = 0.00 s. Subsequently, the *γ*_*V*_ in the pump rises rapidly, at which time the self-priming pump enters the shock exhaust stage. Then at *t* = 2.05 s, the *γ*_*V*_ in the pump chamber goes to the maximum value, which is about 47.79 %. Combined with Fig. 11, it can be seen that at about *t* = 2.50 s and *t* = 6.00 s, corresponding to the self-priming pump entered the rapid exhaust stage and the residual gas discharge stage in the pump, respectively. Finally, at *t* = 11.5 s, the *γ*_*V*_ reaches below 2 %, indicating the completion of the self-priming process. In the whole self-priming process, the first stage is the rapid suction stage, lasting approximately 0.40 s, which accounts for 3.46 % of the total self-priming duration; The second stage is the shock exhaust stage, lasting approximately 2.10 s, accounting for 18.18 % of the total self-priming duration; The third stage is the rapid exhaust stage, lasting approximately 3.50 s, accounting for 30.30 % of the total self-priming duration; The fourth stage is the pump residual gas discharge stage, lasting approximately 5.55 s, accounting for 48.05 % of the total self-priming duration.

### 3.3 Vortex identification

During the self-priming process, there exists a complex process of repeated changes of gas-liquid two-phase flow in the pump. With the help of the vortex identification method, the distribution area of vortices in the flow field of the self-priming pump can be quickly determined, and information such as the position, size, and intensity of the vortices can be provided, which can better explore the internal flow and energy transfer during the self-priming process of the pump.

The *Q*-criterion is a classical method in vortex identification and is based on calculating the velocity gradient tensor. The characteristic equation for the local velocity gradient tensor of an incompressible flow in a centrifugal pump is [23,24]:

(8) $$\lambda^{3}+P\lambda^{2}+Q\lambda +R=0$$

Where: *P*, *Q* and *R* are three independent invariants of the velocity gradient of the following form:

(9) $$\left\{ \begin{array}{c} P=S_{ii}=0\\ Q=\left(\Omega_{ij}\Omega_{ji}-S_{ij}S_{ji}\right)/2\\ R=-\left(S_{ij}S_{jk}S_{ki}+3\Omega_{ij}\Omega_{jk}S_{ki}\right)/3 \end{array}\right.$$

Where: $S_{ij}=\left(\partial u_{i}/\partial x_{j}+\partial u_{j}/\partial x_{i}\right)/2$ is the strain tensor, $\Omega_{ij}=\left(\partial u_{i}/\partial x_{j}-\partial u_{j}/\partial x_{i}\right)/2$ is the vortex tensor.

**Fig. 17:**
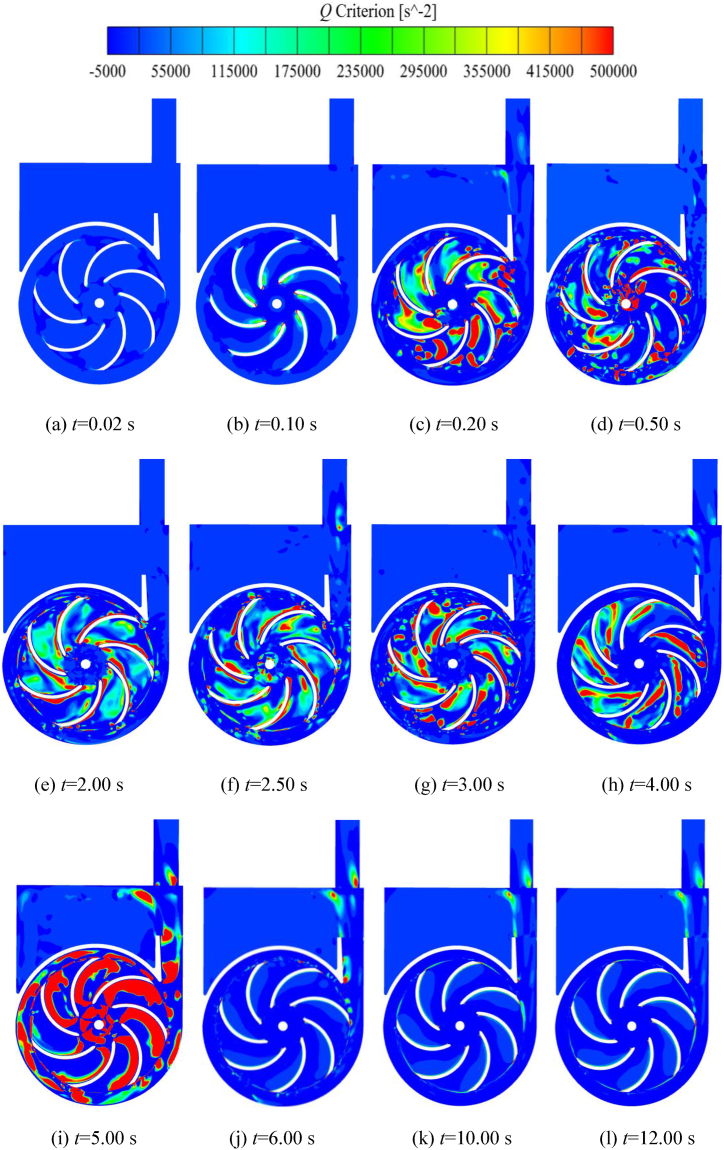
Distribution of vortex in the cross section of the impeller during the self-priming process.

Hunt et al. [25,26] proposed to define the region of $Q=\left(\Omega_{ij}\Omega_{ji}-S_{ij}S_{ji}\right)>0$ as a vortex, the value of *Q* reflects the relationship between the movement and deformation of the fluid microelement in a physical sense. The regions with higher *Q* values have more intense rotational motion, and the position of the vortex is closer to the vortex core. Fig. 17 shows the evolution of the vortex distribution over time in the middle section (xoy plane) of the impeller during the self-priming process. It can be seen that within *t* = 0.02 s, there are very few vortices, which are distributed at the outer edge of the back of the blade. At *t* = 0.10 s, as the impeller gradually accelerates its rotation, there is a noticeable vortex distribution near the inlet of the blades. The *Q* value and distribution area on the back of the blades are larger relative to the working surface, and the location of the largest *Q* value is at the inlet of the blades. At the same time, there is very little vortex at the outer edge of the blades, and the *Q* value is small. Before *t* = 0.10 s, the pump chamber is completely occupied by the liquid, there is no complex process of gas-liquid two-phase mixing, and the vortex distribution is approximately centrosymmetric. When *t* = 0.20 s, there is a very obvious vortex distribution in the impeller channel, and the *Q* value is very large. Most of the vortex is distributed in the impeller channel, and a very small portion exists in the volute. This is mainly because the gas phase has entered the impeller channel at this time, and the flow is more chaotic and complex. From Fig. 17(d) and (e), it can be seen that there is a large distribution of vortex within both impeller and volute domains. At *t* = 0.50 s, the vortex is mainly distributed in the impeller channel with a large *Q* level, while it is relatively less in the volute. During the latter part of the shock exhaust stage and the rapid exhaust stage (2.00 s < *t* < 4.00 s), the liquid in the inlet pipe enters the impeller, and the gas-liquid phases coexist in the impeller channel. The vortex approximately shows a centrosymmetric distribution, and the regions with larger *Q* values are basically located in the impeller channel and the clearance between the impeller and the volute. At *t* = 5.00 s, combined with Fig. 14, Fig. 15, it can be seen that the gas in the front pump chamber starts to be sucked into the impeller along with the liquid, causing the flow to become unstable. As a result, a large number of vortex appear in the impeller domain, and the *Q* value is very high. After *t* = 6.00 s, the impeller and volute domains are full of liquid, and the single-phase flow loss is low. A small amount of vortex exists on the back of the blades, on the outer edge of the impeller, in the gas-liquid separation chamber, and at the pump outlet, while the vortex inside the volute gradually disappears.

**Fig. 18:**
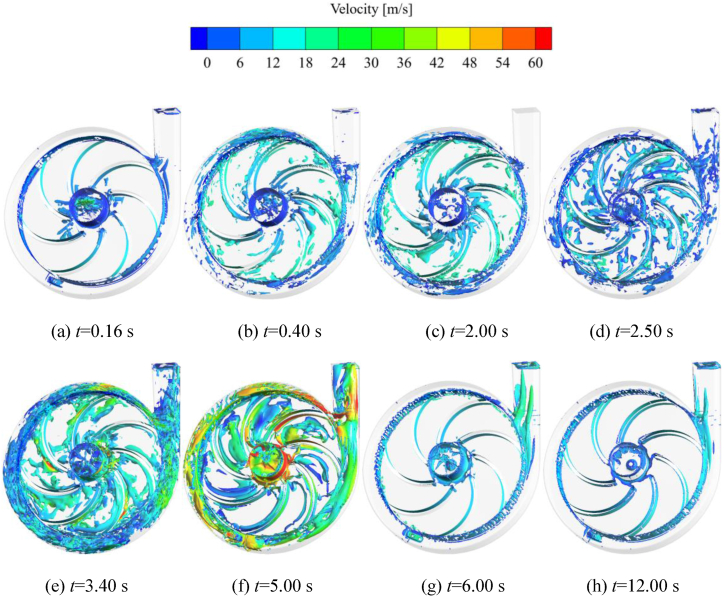
Evolution of the vortex in the impeller and volute during self-priming process.

The vortex core is an essential manifestation of the vortex flow, which can more visually demonstrate the shape, location, intensity, and influence range of the vortex. Fig. 18 shows the evolution of the vortex core in the impeller and volute domains during the self-priming process. The vortex core is extracted from the flow field using a *Q*-criterion with a given threshold value. The color of the vortex core is demonstrated in terms of the velocity of the flow field, and the threshold value of the *Q* criterion is set to 661,560 s^−2^. It can be seen that when *t* = 0.16s, the distribution of vortex core in the impeller and volute domains is less overall. The main distribution of the vortex core is on the backside of blades and at the outlet of the impeller, with a few present at the reflux hole and the tongue of the volute, and the velocity is relatively low. This is because the self-priming pump is in the rapid suction stage, and the impeller has not yet reached its full speed. The gas has just entered the impeller and the internal flow is smooth. When *t* = 0.40 s, compared with *t* = 0.16 s, a small amount of vortex core is in the impeller channel. At the same time, the vortex core at the outer edge of the impeller, the blade wall, the volute domain and the tongue gradually increase. This is because the gas sucked into the impeller has been thrown to the outer edge of the impeller to mix with the liquid. Since the outer edge of the impeller and the volute are the main places for gas-liquid mixing, the flow is complicated and vortex core is generated. Fig. 18(d)–(f) indicate that the self-priming pump is in the rapid exhaust stage, the liquid in the inlet pipe enters the impeller, and the gas in the impeller channel is gradually discharged into the volute for gas-liquid mixing. The energy loss within the flow field of the pump is significant, and the number of vortex in the impeller and volute domains is large with high velocity overall. From *t* = 3.40 s and *t* = 5.00 s, it can be seen that the vortex with larger flow velocity is mainly distributed in the impeller inlet, the middle position of the blade wall, the reflux hole and the whole channel of the volute. After *t* = 6.00 s, the impeller and volute are basically occupied by the liquid phase, and the vortex in the flow channel of the impeller and volute is sharply reduced. However, there is still a small number of the vortex distributed near the impeller inlet, the blade wall, the reflux hole, and the tongue of the volute. As time goes by, the vortex at the impeller inlet, the tongue of the volute and the outlet section show a tendency to decrease.

### 3.4 Entropy production rate analysis

Entropy production refers to the inevitable effects caused by irreversibility in a process, where the loss of mechanical energy is converted into internal energy, resulting in an increase in entropy. The numerical simulation of the self-priming pump is based on the Reynolds time-averaged equation. For turbulent motion, the entropy production rate (*EPR*) can be divided into two parts, which are caused by the average velocity and the transient pulsation velocity, respectively. The total entropy production rate ${\dot{S}}^{\prime \prime \prime }$ per unit volume can be expressed as [27]:

(10) $${\dot{S}}^{\prime \prime \prime }={\dot{S}}_{D}^{\prime \prime \prime }+{\dot{S}}_{D^{\prime }}^{\prime \prime \prime }$$

where: ${\dot{S}}_{D}^{\prime \prime \prime }$ is the direct dissipation term caused by the mean velocity, and ${\dot{S}}_{D^{\prime }}^{\prime \prime \prime }$ is the turbulent dissipation term caused by the transient pulsation velocity.

The expression for the direct dissip ation term ${\dot{S}}_{D}^{\prime \prime \prime }$ and the turbulent dissipation term ${\dot{S}}_{D^{\prime }}^{\prime \prime \prime }$ is:

(11) $${\dot{S}}_{D}^{\prime \prime \prime }=\frac{2\mu }{T}\left[{\left(\frac{\partial \bar{u}}{\partial x}\right)}^{2}+{\left(\frac{\partial \bar{v}}{\partial y}\right)}^{2}+{\left(\frac{\partial \bar{w}}{\partial z}\right)}^{2}\right]+\frac{\mu }{T}\left[{\left(\frac{\partial \bar{v}}{\partial x}+\frac{\partial \bar{u}}{\partial y}\right)}^{2}+{\left(\frac{\partial \bar{w}}{\partial x}+\frac{\partial \bar{u}}{\partial z}\right)}^{2}+{\left(\frac{\partial \bar{v}}{\partial z}+\frac{\partial \bar{w}}{\partial y}\right)}^{2}\right]$$

(12) $${\dot{S}}_{D^{\prime }}^{\prime \prime \prime }=\frac{2\mu_{eff}}{T}\left[{\left(\frac{\partial u^{\prime }}{\partial x}\right)}^{2}+{\left(\frac{\partial v^{\prime }}{\partial y}\right)}^{2}+{\left(\frac{\partial w^{\prime }}{\partial z}\right)}^{2}\right]+\frac{\mu_{eff}}{T}\left[{\left(\frac{\partial v^{\prime }}{\partial x}+\frac{\partial u^{\prime }}{\partial y}\right)}^{2}+{\left(\frac{\partial w^{\prime }}{\partial x}+\frac{\partial u^{\prime }}{\partial z}\right)}^{2}+{\left(\frac{\partial v^{\prime }}{\partial z}+\frac{\partial w^{\prime }}{\partial y}\right)}^{2}\right]$$

where: μ represents the dynamic viscosity; $\bar{u}$、 $\bar{v}$、 $\bar{w}$ is the time-averaged velocity; $u^{\prime }$、 $v^{\prime }$、 $w^{\prime }$ is the pulsation velocity; *T* is the temperature (set to a constant value of 293 K); $\mu_{eff}=\mu +\mu_{t}$ is the effective dynamic viscosity, where $\mu_{t}$ is the turbulent dynamic viscosity.

Here ${\dot{S}}_{D}^{\prime \prime \prime }$ can be solved directly by numerical computation and ${\dot{S}}_{D^{\prime }}^{\prime \prime \prime }$ cannot be solved directly by numerical computation.

For the turbulent dissipation term caused by transient pulsating velocity, Kock et al. [28,29]. Proposed a simpler algorithm to calculate it, as in Eq. (13).

(13) $${\dot{S}}_{D^{\prime }}^{\prime \prime \prime }=\frac{\rho \varepsilon }{T}$$

where: *ρ* is the density of the medium, *ε* is the dissipation rate of turbulent kinetic energy.

Due to the strong wall effects and significant time-averaged terms, the equation for calculating entropy production near the wall is as follows:

(14) $$S_{pro,W}=\int_{S}\frac{\vec{\tau }\cdot \vec{v}}{T}dS$$

Where: τ is the wall shear stress, Pa; *S* is the area, m^2^; *v* is the velocity near the wall, m/s.

Therefore, the total entropy production in the entire computational domain of the system is calculated as:

(15) $$S_{pro\;}=\int_{V}{\dot{S}}_{D}^{\prime \prime \prime }dV+\int_{V}{\dot{S}}_{D^{\prime }}^{\prime \prime \prime }dV+\int_{S}\frac{\vec{\tau }\cdot \vec{v}}{T}dS$$

**Fig. 19:**
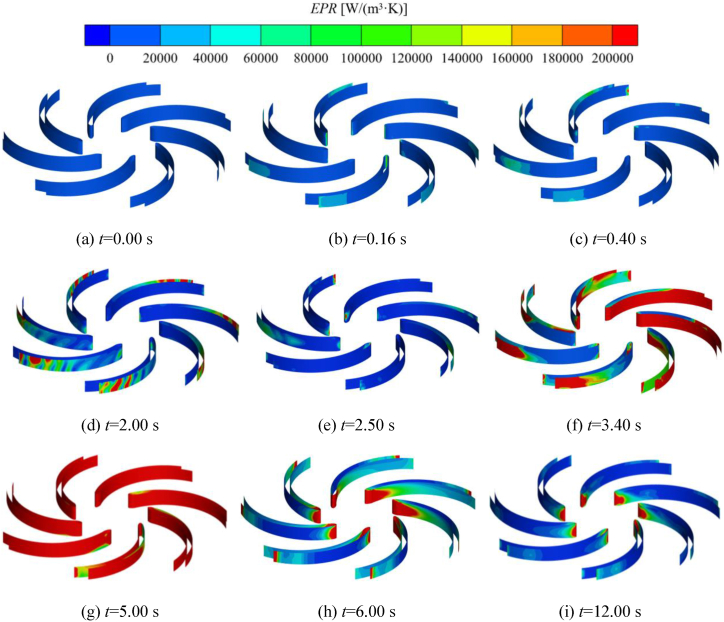
Distribution of entropy production rate on the blade wall during the self-priming process.

The self-priming pump is usually accompanied by complex gas-liquid two-phase flow during the self-priming process, which leads to the increase of entropy production. Fig. 19 shows the distribution of entropy production on the wall of blades during the self-priming process. From Fig. 19(a)–(c), it can be noticed that the region of entropy production rate and the value of it is small overall, with only a few present at the inlet of the blade and at the end of the working surface of the blade. This is because at the beginning of the self-priming pump startup, the impeller chamber is full of liquid, and the rotational speed is slower, so the flow state is relatively gentle, and the loss is less. Over time, the gas in the impeller is discharged to the outer edge of the impeller for mixing with the liquid, resulting in regions of higher losses, and the distribution of the entropy production rate increases gradually at the outer edge of the working surface of the blade. Combined with Fig. 19(d)–(g), it can be seen that the process of gas-liquid mixing at the outer edge of the impeller recurs throughout the shock exhaust stage. Therefore, when 0.40 s < *t* < 2.50 s, the majority of high entropy production rate regions are still located at the outer edge of the impeller and the blade working surface, with a small presence at the impeller inlet. During the rapid exhaust phase, when 2.50 s < *t* < 6.00 s, the high entropy production rate regions are primarily located at the blade inlet and the outer edge of the blade. At *t* = 3.40 s and *t* = 5.00 s, the distribution of entropy production rate is most significant. This is mainly due to the mixing of residual gas in the inlet S-pipe and the front pump chamber with the liquid entering the impeller. The two-phase flow inside the impeller is complex, leading to a mixed flow state at the blade inlet and outer edge, which produces more losses. At *t* = 6.00 s, the impeller is full of liquid phase, and the regions with higher losses are basically distributed at the inlet of the blades and the outer edge of the impeller. This is mainly due to the domain of the impeller always rotating at a high speed, and the motion of the liquid entering and exiting the impeller undergoes significant changes, resulting in certain losses.

**Fig. 20:**
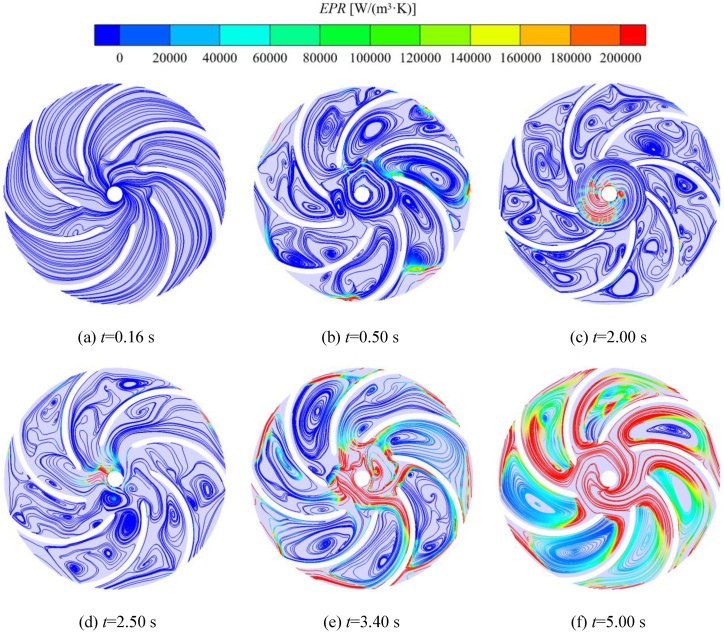
Streamlines of the relative velocity of the impeller during the self-priming process.

Fig. 20 shows the distribution of relative velocity streamlines in the middle cross-section of the impeller during self-priming, the color of the streamlines is demonstrated in terms of the distribution of entropy production rate in the impeller. Combining Fig. 20, Fig. 13, in the initial stage of the self-priming process, the gas in the inlet pipe enters the impeller. It can be seen that the flow separation phenomenon exists at the gas-liquid interface and the entropy production rate is present very little at this point. Fig. 20(b)–(d) are in the shock exhaust stage, the majority of the impeller domain is occupied by the gas, the flow is extremely turbulent, there are a large number of vortex in each flow channel. In this stage, the gas is mixed with a small amount of liquid in the inlet S-pipe and the front pump chamber when it is sucked into the impeller, and the process of gas-liquid two-phase mixing always exists at the outer edge of the impeller, and there is always a gas-liquid two-phase mixing process at the outer edge. Therefore, there are local high entropy production regions at the impeller inlet and outer edge. When at *t* = 3.40 s and *t* = 5.00 s, the distribution region and value of entropy production increases significantly at the inlet and the outer edges of the impeller and there are large sized vortex in each flow channel.

### 3.5 Stabilization analysis

In the process of self-priming, the mixing of gas and liquid phases mainly occurs in domains of the impeller and the volute, resulting in unsteady flow conditions, which causes the loss of energy. Dou Huashu et al. [30,31] proposed the energy gradient theory for analyzing the flow stability and problems related to turbulent turning in centrifugal pumps. According to the energy gradient theory, the calculation equation of the energy gradient parameter can be expressed as:

(16) $$K=\frac{\partial E}{\partial n}{\left(\frac{\partial H}{\partial s}\right)}^{-1}=\frac{\frac{\partial p}{\partial n}+\rho U\frac{\partial U}{\partial n}}{\frac{\mu_{t}}{U}{\left(\frac{\partial U}{\partial n}\right)}^{2}-\frac{2\mu_{h}}{\rho U^{2}}\cdot \frac{\partial U}{\partial n}\cdot \frac{\partial p}{\partial n}+\frac{\mu_{t}}{\rho^{2}U^{3}}{\left(\frac{\partial p}{\partial n}\right)}^{2}}$$

(17) $$E=p+\frac{1}{2}\rho \left(u^{2}+v^{2}+w^{2}\right)$$

where: *E* is the total pressure of the fluid, *H* is the loss of mechanical energy along the streamlined direction, *U* is the characteristic velocity, *n* is the normal direction of the fluid flow, *s* is the streamlined direction of the fluid flow, *p* is the static pressure, *ρ* is the density of the fluid, *u* is the velocity component in the x-axis direction, *v* is the velocity component in the y-axis direction, and *w* is the velocity component in the z-axis direction.

**Fig. 21:**
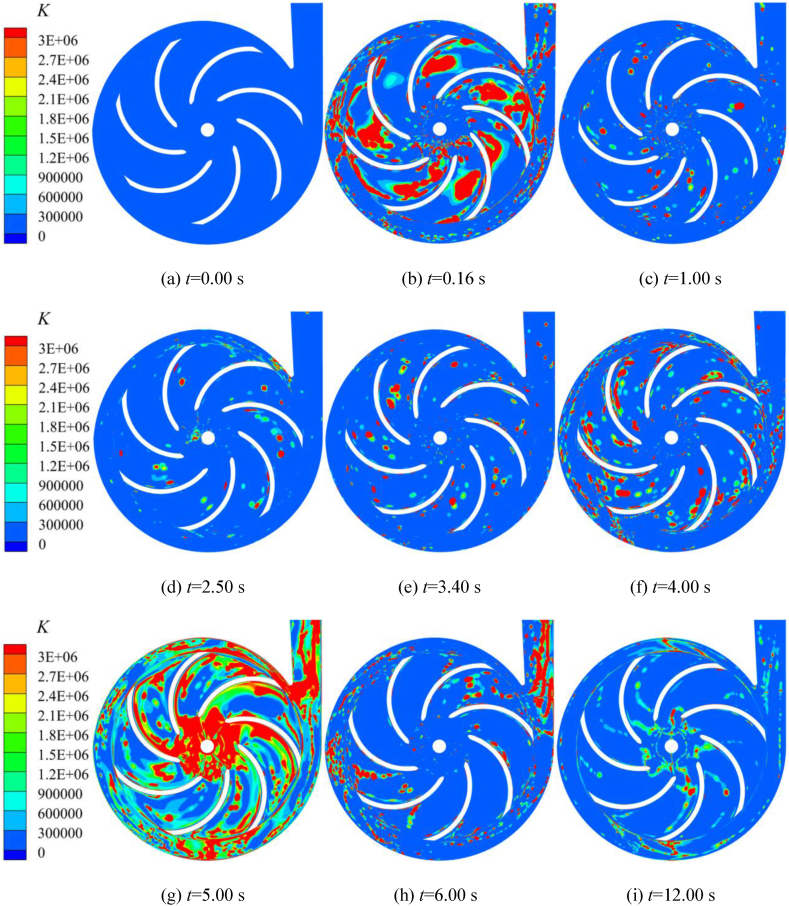
Distribution of the energy gradient function *K* of the cross-section in the impeller and volute during the self-priming process.

In order to investigate the situation of the energy gradient during the startup of the pump, the evolution of the energy gradient *K* in the impeller and the volute domains at different moments are shown in Fig. 21. At *t* = 0.16 s, there are large destabilization regions, which are mainly distributed in the impeller channel, the bottom of the impeller inlet, and in the volute channel. The regions of high *K* value in the volute domain are mainly concentrated in the Ⅰ, Ⅲ and Ⅳ sections. This is because the gas in the inlet pipe just entered the impeller at this time, the gas-liquid two-phase coexistence in the impeller channel, the flow state is complex, coupled with the impeller is accelerating the rotation at this moment, so there is a large area of instability and significant energy loss. At *t* = 1.00 s, the self-priming pump enters the shock exhaust stage, the impeller rotates with constant speed, and the vast majority of the liquid in the impeller and volute domains is gradually discharged from the volute outlet. The region of destabilization is significantly reduced compared to the rapid suction stage. The high *K* value region is mainly distributed in the impeller domain and less in the volute domain. Fig. 21(d)–(h) show that the high *K* value region in the impeller basin shows a gradually increasing trend during the rapid exhaust stage. At *t* = 3.40 s and *t* = 5.00 s, the residual gas in the inlet S-pipe and part of the gas in the front pump chamber is sucked into the impeller. Therefore, the flow instability occurs again, and the high *K* value region in the impeller and volute basins gradually increases again, with *t* = 5.00 s being the most obvious. Fig. 21(h) and (i) show that in the pump residual gas discharge stage, with the discharge of the gas in the volute and impeller domains, the energy loss generated by the pure liquid-phase flow is small, so the high *K* value region gradually decreases. At *t* = 6.00 s, because there is still some gas remaining in the left wall of the volute outlet section that has not been discharged, it is mixed with the liquid rapidly discharged from the volute, so the volute outlet section appears regions with high *K* value. At the same time, there is also a part of high *K* value region around the Ⅰ, Ⅳ, and Ⅷ sections of the volute and the corresponding impeller channel; At *t* = 12.00 s, the high *K* value region is mainly concentrated in the inlet and outlet of the blade, and nearby the Ⅱ and Ⅵ sections of the volute.

## 4 Conclusion

In order to investigate the evolution of gas-liquid two-phase flow and energy loss inside the pump during the startup process of the self-priming pump, this article builds a circulatory piping calculation system of the self-priming pump, which includes the self-priming pump, tank, valve, inlet pipe and outlet pipe. Only the top surface of the storage tank is set to atmospheric pressure, and the whole calculation process realizes the self-coupling solution without providing other boundary conditions. The calculation process takes into account the accelerated rotation and constant-speed rotation states of the impeller after the self-priming pump is started, which fully reflects the actual situation and accurately represents the entire self-priming process of the pump. As a result, more accurate results are obtained. The conclusions drawn from the analysis are as follows:

(1) The self-priming process of this self-priming pump can be roughly divided into four stages: The first stage is the rapid suction stage, accounting for 3.46 % of the total self-priming time; The second stage is the shock exhaust stage, accounting for 18.18 % of the total self-priming time; The third stage is the rapid exhaust stage, accounting for 30.30 % of the total self-priming time; The fourth stage is the pump residual gas discharge stage, accounting for 48.05 % of the total self-priming time. It can be seen that the proportion of each stage in the total self-priming time shows an increasing trend in sequence.
(2) In the vertical section of the inlet pipe, the process of the water level rising shows a pattern of initially slow and then fast. Eventually, the water level reaches the horizontal section of the inlet pipe and enters the pump. In the early stage of the self-priming process, the water level rises slowly. This is because the water body in the whole system is initially in a stationary state, with a large inertia. And the rotational speed of the impeller in the early stage of the self-priming process is low, so the pressure drop at the impeller inlet is not obvious, and the suction ability is poor.
(3) In the shock exhaust stage, the average gas volume fraction in the volute and the gas content at the volute outlet are overall lower than that at the impeller inlet. This is mainly due to the liquid in the inner wall of the volute cannot be discharged in time in this stage; And due to the negative pressure generated by the rotation of the impeller, part of the liquid in the lower part of the gas-liquid separation chamber will enter the bottom of the volute through the reflux hole.
(4) In the self-priming process, there are four time nodes in the results shown: the gas-liquid two-phase flow inside the pump is intense, the flow inside the pump is prone to be unsteady, and the energy loss is large. *t* = 0.16 s, the gas in the inlet S-pipe starts to enter the impeller and mix with the liquid phase; *t* = 2.50 s, the liquid in the inlet pipe enters the impeller and mixes with the gas phase; *t* = 3.40 s, part of the gas remaining in the inlet S-pipe and in the clearance of the front chamber is sucked into the impeller; *t* = 5.00 s, part of the gas in the clearance of the front pump chamber is sucked into the impeller.
(5) In the whole self-priming process, the inlet and outer edge of the impeller is always the area of high energy loss.

## Data accessibility

The data used to support the findings of this study are available from the corresponding author upon request.

## CRediT authorship contribution statement

**Yu-Liang Zhang:** Writing – original draft, Conceptualization. **Kai-Yuan Zhang:** Software. **Yan-Juan Zhao:** Software. **Jin-Fu Li:** Validation. **Shao-Han Zheng:** Validation.

## Declaration of competing interest

The author(s) declared no potential conflicts of interest with respect to the research, authorship, and/or publication of this article.
